# Conservation Planning for Promoting Ecosystem Service Provisioning Outside Protected Area Networks

**DOI:** 10.1002/ece3.72576

**Published:** 2025-11-29

**Authors:** Florence Godfrey Tarimo, Francis Moyo, Claire Kelly, Linus Kasian Munishi

**Affiliations:** ^1^ Department of Sustainable Agriculture, Biodiversity and Ecosystem Management The Nelson Mandela African Institution of Science and Technology Arusha Tanzania; ^2^ Department of Geography and Economics Mkwawa University College of Education Iringa Tanzania; ^3^ School of Geography, Earth and Environmental Sciences University of Plymouth Plymouth UK

**Keywords:** areas outside protected area networks (OPAN areas), ecosystem‐based conservation (EBC) framework, restoration, valuing ecosystem services

## Abstract

Among factors that contribute to global biodiversity loss, habitat loss through unsustainable land use and land cover changes has gained prominence, with impacts being exacerbated by increasing human populations. Establishing protected area networks (PANs) is strongly advocated by national and international mechanisms, such as the Convention on Biological Diversity (CBD), as a primary strategy to guide biodiversity conservation and management; however, this can undermine conservation efforts outside protected areas. Understanding how people and biodiversity overlap and interact outside protected area networks (OPAN areas) is essential for setting realistic, sustainable targets to guide biodiversity conservation and ecosystem service provision beyond PAN. However, how OPAN areas can sustain or enhance ecosystem services (ESs) through improved conservation and management remains unassessed. We applied a novel ecosystem‐based conservation (EBC) framework, using data from Tanzania, to assess how biodiversity and land use/land cover (LULC) types shape the monetary value of selected ESs in OPAN areas, and in future scenarios where restoration of priority wildlife corridors is implemented by 2030. Across the six ecosystems and four LULC types assessed, waterbody delivered the highest ES value (US$12.8 billion) through water provision and flood control. OPAN areas in miombo woodland also yielded high value (US$12.4 billion), with 46% from flood control and 54% from water provision and carbon storage. ES values varied across OPANs, mainly driven by relative size and degradation level. Restoring 197,497 ha of degraded land within 53 prioritised wildlife corridors in Tanzania could generate up to US$62.8 million annually in ESs under full restoration, and US$31.4 million under partial restoration, with carbon storage contributing over 90% of total value. Policy implications: The spatial heterogeneity and ES overlap observed highlight the need to integrate OPANs into the EBC framework, linking local restoration gains with national policies and global biodiversity frameworks.

## Graphical abstract

We assessed how biodiversity and land use/land cover (LULC) interact to shape ecosystem service (ES) values in areas outside protected area networks (OPANs) across six Tanzanian terrestrial ecosystems. Using a novel ecosystem‐based conservation (EBC) framework, we show that waterbody LULC and the miombo woodland ecosystem deliver high ES values, and that restoring degraded OPAN areas could generate additional benefits. Our findings highlight the critical role of OPANs in national biodiversity conservation and restoration planning.

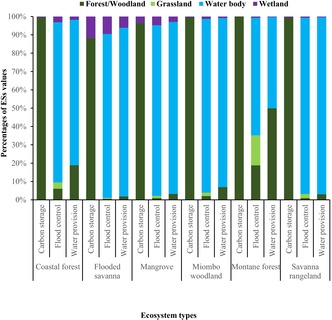

## 1 Introduction

Global ecosystems offer numerous benefits to society, often referred to as ecosystem services (ESs), which encompass the conditions, processes, and elements of the natural environment that support human well‐being (Fisher et al. 2009; MEA 2005; Potschin and Haines‐Young 2016). ESs are, therefore, priceless benefits derived from natural systems, including the resources and processes that sustain life on the planet (Hussein 2021; MEA 2005; Sandifer et al. 2015). At a global scale, terrestrial ecosystems such as forests, wetlands, woodlands, and grasslands provide a range of ESs crucial for sustaining the livelihoods of human communities inhabiting those ecosystems (Fisher et al. 2011; Koko et al. 2020; Ligate et al. 2017; Munishi et al. 2010; Nelson et al. 2010). Despite the potential to provide ESs, these ecosystems are increasingly under threat (Zhongming et al. 2019), particularly in regions where natural resources are essential for supporting livelihoods (Egoh et al. 2012; Zhongming et al. 2019).

Protected area networks (PAN) within these ecosystems are central to conserving biodiversity and supporting ES provision (Hummel et al. 2019; Maes et al. 2012; Vačkář et al. 2016), but they are often perceived as the sole contributors to ES provision (He et al. 2018; Hummel et al. 2019; Ninan and Kontoleon 2016). This perception neglects the potential for ESs in areas outside protected area networks (OPAN areas), particularly when conservation practices are optimised. Clarifying this misconception and supporting conservation efforts in both PAN and OPAN areas at the ecosystem level is critical for an inclusive approach to the sustainable management of ecosystems. Although a significant proportion of important terrestrial ecosystems remains unprotected, hence exist as OPAN areas (Dinerstein et al. 2017), these areas do not necessarily need to be incorporated into PAN. Rather, they require evidence‐based guidance to sustain biodiversity conservation and optimise ES supply. To address this, we apply an Ecosystem‐based Conservation (EBC) framework, which integrates biodiversity conservation with ecosystem service provision and sustainable land management. The EBC framework provides a pragmatic approach for guiding restoration interventions and balancing conservation efforts between PAN and OPAN areas, thereby optimising the supply of ESs. This aligns with the Kunming‐Montreal Global Biodiversity Framework (Global Biodiversity Framework 2022), particularly targets on participatory spatial planning (target 1), ecosystem restoration (target 2), sustainable species management (target 9), enhancing ecosystem services (target 11), increasing green spaces (target 12), sustainable consumption (target 16), and promoting biodiversity conservation incentives (target 18). It further supports the African Union's Agenda 2063 for sustainable production and consumption (Archer et al. 2018).

Land use/land cover (LULC) types within ecosystems shape the structure, function, and dynamics of ecosystems, influencing their potential to provide ESs (Admasu et al. 2023; Belay et al. 2022; Marino et al. 2023). Quantifying ES values across LULC types in OPAN areas reveals their contribution to biodiversity conservation and ES provisioning, guiding policy and practical restoration strategies at the ecosystem level (Perez‐Verdin et al. 2016). Economic valuation of ESs highlights their potential, fosters restoration, and promotes sustainable management practices in OPAN areas.

Although approaches to ES valuation vary widely in spatial scale and methods (monetary and non‐monetary) (Costanza et al. 1997; Meraj et al. 2022; Pandeya et al. 2016; Turner et al. 2016), most ES assessments focus on broad‐scale or protected‐area contexts. There remains a critical gap in ecosystem‐level economic valuations of ESs within unprotected areas. This study focuses on OPAN areas across Tanzania's terrestrial ecosystems, which support both biodiversity and local livelihoods. Despite calls for inclusive ecosystem‐based conservation strategies, little is known about how different LULC types within OPAN areas contribute to ES monetary value or their role in meeting national and global conservation targets.

To address this gap, we present a novel EBC framework for the economic evaluation of ESs within OPAN areas. We mapped the spatial coverage of various LULC types across OPAN areas in different ecosystems and examined how biodiversity and LULC influence the monetary value of selected ESs. We also assessed alternative scenarios in which restoration is carried out in priority wildlife corridors by 2030. While the framework has broader relevance, this study presents a data‐driven case from Tanzania to demonstrate its practical application.

## 2 Materials and Methods

### 2.1 Study Area and Studied Ecosystems

**FIGURE 1:**
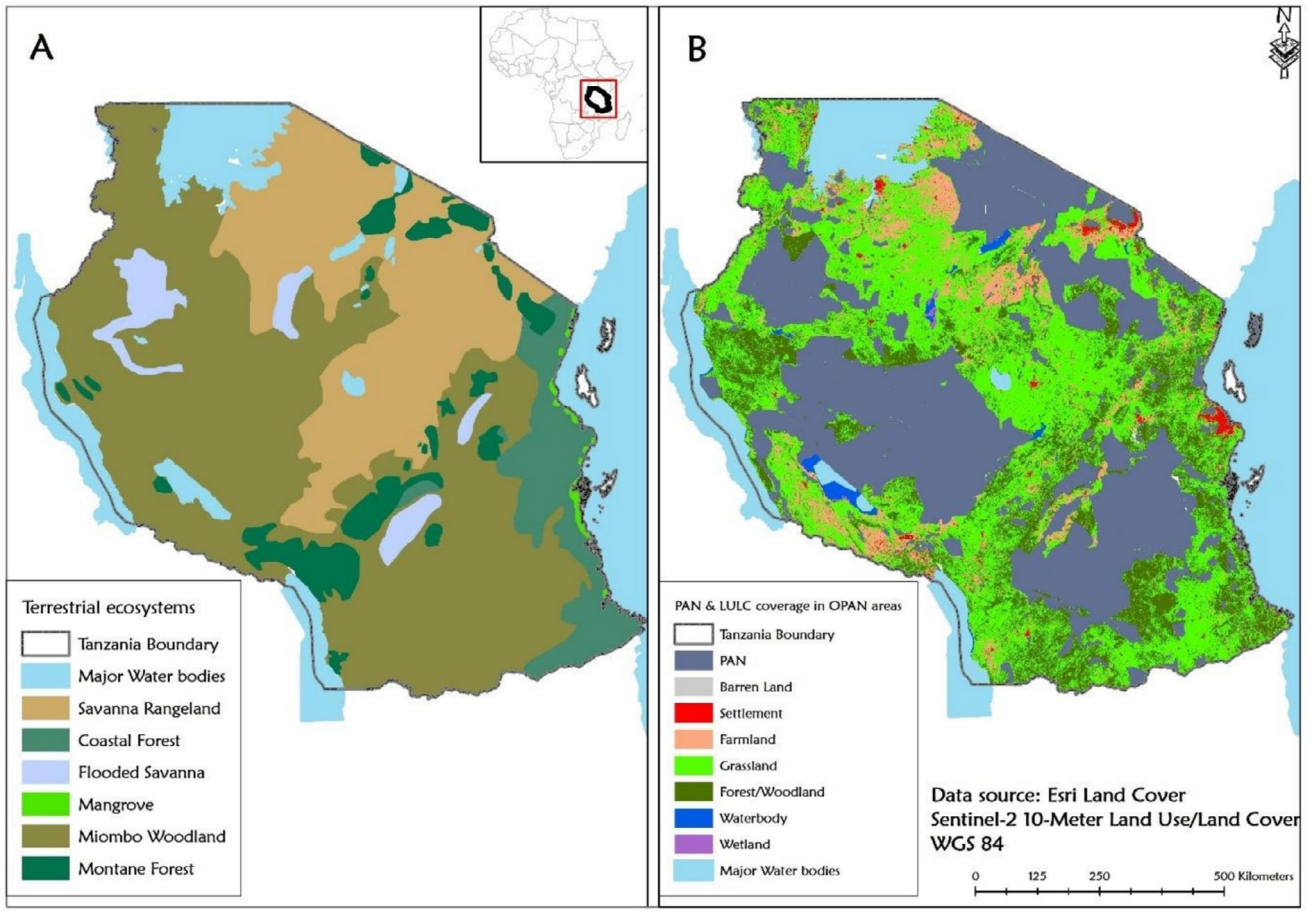
Spatial distribution of terrestrial ecosystems and LULC in OPAN areas. (Panel A) Major terrestrial ecosystem types in Tanzania and (Panel B) LULC types within OPAN areas.

We conducted our study in the OPAN areas of the eight major terrestrial ecosystems in mainland Tanzania (UNEP 2016). Tanzania is located between 29° E and 41° E and 1° S and 12° S and covers an area of 945,100 km^2^ (Mauya et al. 2019). The country exhibits diverse bioclimatic and topographic zones, ranging from arid regions with less than 400 mm of annual rainfall to humid areas with over 2000 mm and temperatures from 5.3°C to 33.1°C. These ecosystems included montane forest, recognised for their climatic stability and high levels of endemic species, and provision of key ESs (Burgess et al. 2007); and adjacent high‐altitude moorlands support specialised afro‐alpine flora adapted to harsh conditions (Andreas 2002). Flooded savannas, such as the Kilombero Valley, feature seasonally inundated grasslands and swamp mosaics that sustain wetland‐dependent species and regulate regional hydrology (Andrew et al. 2012). Grasslands dominated by herbaceous vegetation support herbivores and large carnivores (Smith et al. 2020), whereas semi‐arid acacia savannas maintain diverse grazing systems and large mammal populations (Owen‐Smith et al. 2019). Miombo woodland, dominated by *Brachystegia* and *Julbernardia*, are ecologically diverse and vital for rural livelihoods (Ribeiro et al. 2020). Coastal forests along the Indian Ocean, though fragmented, host high species richness and endemism (Burgess et al. 1998), and mangroves composed of diverse species that include *Rhizophora*, *Avicennia*, and *Sonneratia* provide shoreline protection and fish nursery habitats (Bosire et al. 2016). For this study we modified the eight major terrestrial ecosystem categories in Tanzania (UNEP 2016) by merging grassland and acacia savanna into the category of ‘savanna rangeland ecosystem’. This is because the two form a continuous ecological system with shared species and disturbance regimes, and are therefore classified as such in most scientific literature work (Scheiter et al. 2019; Selemani and Sangeda 2019). Montane forest and moorland were also combined into a single ‘montane forest ecosystem’ category. While moorlands occur at higher elevations, they are commonly regarded as ecological extensions of montane forests, forming a continuous altitudinal gradient (Gebrezgiher et al. 2022; Mbije et al. 2007). Accordingly, most literature treats moorland as part of the broader montane forest ecosystem. This brought the total number of ecosystem types included in this study to six: montane forest, savanna rangeland, coastal forest, flooded savanna, miombo woodland and mangrove (Figure 1A).

### 2.2 Establishing the Sizes of Different LULC Types in OPAN Areas in Different Ecosystems

We assessed LULC types within OPAN areas across terrestrial ecosystems in Tanzania (Figure 1B). The LULC dataset used in this study was derived from the 2022 ESRI Sentinel‐2 imagery at 10 m resolution in the Universal Transverse Mercator (UTM) WGS84 coordinate system (Karra et al. 2021). The dataset classified LULC into seven categories: barren land, settlement, farmland, grassland, forest/woodland, water body, and wetland, as described in Table S1. The raster data were processed and analysed using ArcGIS 10.4 and ArcGIS Pro 3.2. The study area, defined using a polygon shapefile of Tanzania's ecosystems, was clipped to extract LULC data specific to OPAN areas using the Clip Raster by Mask tool. The extracted data were projected into the study's coordinate system. The protected areas defined as National Parks, the Ngorongoro Conservation Area, Nature Reserves, Game Reserves, Forest Reserves, Wildlife Management Areas, Game Controlled Areas, and Game Open Areas, were excluded from the map to focus solely on OPAN areas. LULC classification accuracy was enhanced through post‐processing steps, including majority filtering and clumping, to reduce errors in the map. The LULC types and sizes for each ecosystem were quantified in hectares (ha) and analysed for spatial attributes within OPAN areas. These outputs were used for ES economic valuation. The overall accuracy of the LULC classification was 85%, ensuring reliable data for subsequent analysis. In total, OPAN areas accounted for a substantial proportion of each ecosystem, ranging from 45% in the flooded savanna ecosystem to 92% in the Mangrove ecosystem, as shown in Table 1.

**TABLE 1:** Ecosystem sizes and estimated OPAN coverage in hectares across terrestrial ecosystems.

Ecosystem type	Ecosystem size (ha)	OPAN areas (ha)
Miombo woodland	48,494,200	27,156,752
Coastal forest	6,962,400	5,361,048
Montane forest	5,978,200	4,113,002
Savanna rangeland	22,977,700	13,970,442
Mangrove	508,400	467,728
Flooded savanna	3,268,000	1,470,600

### 2.3 Estimating the Monetary Values for Selected ESs

Using the Google Scholar search engine, we conducted an opportunistic literature survey to identify potential ESs across global terrestrial ecosystems. We found that literature classifies these ESs differently (Table S2), and none is universally accepted (Hummel et al. 2019). Flood control ES, for instance, were identified as a hydrological balance in some cases (Kaur 2006), in others, as coastal protection (Arkema et al. 2023), and as flood regulation (Schmidt et al. 2019). We then contextualised these services by adopting the globally recognised ES classification proposed by Chan et al. (2006), which identifies six major ESs: Carbon storage, water provision, flood control, forage production, outdoor recreation, and crop pollination. Of the six ESs, we selected three ESs: Carbon storage, water provision, and flood control, to evaluate the monetary values of ESs in OPAN areas. We selected these three ESs because of their known and documented monetary values per unit area, and their locally perceived potential to deliver tangible benefits to local communities (Dokken et al. 2014; Jacob 2014; Lopa et al. 2012; Zella and Kitali 2024). The Supporting Information document (Table S2) provides more information on ES types offered by terrestrial ecosystems.

We used a benefit transfer method (Grammatikopoulou et al. 2023; Johnston et al. 2015; Loomis et al. 2014; Richardson et al. 2015) to estimate the annual monetary values of carbon storage, water provision, and flood control ESs in OPAN areas in different ecosystems. We calculated the total annual monetary value of water provision, carbon storage, and flood control for relevant LULC types using a straightforward approach based on the idea that if a per‐hectare monetary value is known and the corresponding area is available, the total value can be obtained through multiplication (i.e., value per hectare (ha) × total area). We adopted annual monetary value coefficients from the most recent published study by Msofe et al. (2020) to estimate values for similar LULC types in the study region, while the calculation method applied was developed independently for this research. ES values were obtained from Msofe et al. (2020), which explicitly adopted and contextualised benefit‐transfer estimates to Tanzanian ecosystems. Although we directly applied the values reported by Msofe et al., this implicitly incorporated multiple foundational studies (Costanza et al. 1997; Kindu et al. 2016; Temesgen et al. 2018). Msofe et al. (2020), “bushland” and “forest” LULC were assigned identical values, and as these classes are often indistinct at the landscape scale, we treated them collectively as forest/woodland. “Water” was classified as waterbody, while “grassland” and “wetland” categories were maintained as reported in Msofe et al. (2020). All monetary values originally reported in 1994 USD were adjusted to 2024 USD using the US Consumer Price Index (CPI), with an inflation factor of 2.1 (CPI 1994 = 150.10 and CPI 2024 = 317.60) to reflect current economic conditions (U.S. Bureau of Labor Statistics 2025). While benefit transfer provides indicative values, using coefficients already contextualised to Tanzania ensures that the estimates are informative for relative comparisons across ecosystems rather than as absolute figures.

#### 2.3.1 Estimating Monetary Values for Carbon Storage

To estimate the annual monetary value of carbon storage, we considered LULC types with carbon sequestration potential, specifically forest/woodland and wetland, within OPAN areas across different ecosystems. The annual monetary value for each LULC type was calculated using the formula: The annual monetary value = *Vc*/ha × *A*_*L*_, where *Vc*/ha represents the adjusted monetary value of carbon storage per hectare, and *A*_*L*_ signifies the area of the specific LULC type ‘L’ (Table 2).

**TABLE 2:** Spatial coverage and estimated monetary values of selected ecosystem services across LULC types, based on Msofe et al. (2020).

a	b	c	d	e	f	g	h	i
Savanna rangeland	Grassland	9,175,200	—	—	—	—	6	57,803,760
	Forest/woodland	1,799,600	468	842,752,680	17	30,233,280	13	22,674,960
	Waterbody	212,500	—	—	4446	944,711,250	11,435	2,429,831,250
	Wetland	14,900	302	4,505,760	273	4,073,645	1126	16,771,440
Miombo woodland	Grassland	16,358,300	—	—	—	—	6	103,057,290
	Forest/woodland	9,341,800	468	4,374,764,940	17	156,942,240	13	117,706,680
	Waterbody	469,400	—	—	4446	2,086,811,580	11,435	5,367,354,300
	Wetland	65,900	302	19,928,160	273	18,016,994	1126	74,177,040
Montane forest	Grassland	2,225,600	—	—	—	—	6	14,021,280
	Forest/woodland	1,276,900	468	597,972,270	17	21,451,920	13	16,088,940
	Waterbody	4800	—	—	4446	21,339,360	11,435	54,885,600
	Wetland	500	302	151,200	273	136,500	1126	562,800
Coastal forest	Grassland	2,577,554	—	—	—	—	6	16,238,590
	Forest/woodland	2,257,639	468	1,057,252,344	17	37,928,335	13	28,446,251
	Waterbody	35,886	—	—	4446	159,538,390	11,435	410,338,467
	Wetland	13,296	302	4,020,710	273	3,629,808	1126	14,965,978
Mangrove forest	Grassland	231,414	—	—	—	—	6	1,457,908
	Forest/woodland	88,291	468	41,346,675	17	1,483,289	13	1,112,467
	Waterbody	9674	—	—	4446	43,007,702	11,435	110,617,353
	Wetland	4962	302	1,500,509	273	1,354,626	1126	5,585,227
Flooded savanna	Grassland	779,200	—	—	—	—	6	4,908,960
	Forest/woodland	388,600	468	181,981,380	17	6,528,480	13	4,896,360
	Waterbody	73,500	—	—	4446	326,758,950	11,435	840,435,750
	Wetland	79,700	302	24,101,280	273	21,758,100	1126	89,710,320

*Note:* a: Ecosystem types; b: LULC types; c: LULC area (ha); d: Adjusted carbon storage value estimates of different LULC types in US$/ Ha; from Msofe et al. (2020); e: Total carbon storage value of water provision (US$/year) per LULC type; f: Adjusted water provision value estimates of different LULC types in US$/ Ha; from Msofe et al. (2020); g: Total monetary value of water provision (US$/year) per LULC type; h: Adjusted flood control value estimates of different LULC types (US$/ha; from Msofe et al. 2020); i: Total monetary value of flood control (US$/year) of different LULC types.

#### 2.3.2 Estimating Monetary Values for Water Provision

Water provision value estimation focused on LULC types with water provision capacity, namely forest/woodland, waterbody, and wetland (Msofe et al. 2020; Rotich et al. 2022). The annual monetary value of water provision for each LULC type was calculated as: Total annual monetary value = *V*_*w*/ha_ × *A*_*L*_, where *V*_*w*/ha_ is the adjusted water provision monetary value per hectare and *A*_*L*_ is the area of the respective LULC type ‘L’ (Table 2).

#### 2.3.3 Estimating Monetary Values for Flood Control

Forest/woodland, grassland, waterbody, and wetland LULC types in OPAN areas in different ecosystems had the potential for flood control ES provision (Msofe et al. 2020; Rotich et al. 2022) and, therefore, were used in the computation of monetary values. The monetary value of flood control for each LULC was computed using the formula; The annual monetary Value = *V*_*f*/ha_ × *A*_*L*_, where *V*_*f*/ha_ is the adjusted flood control monetary value per hectare and *A*_*L*_ is the area of the respective LULC type ‘L’ (Table 2).

### 2.4 Developing Scenarios of LULC Types

We developed a restoration scenario for OPAN areas in Tanzania to 2030, focusing on 53 priority wildlife corridors covering 22.1% of OPAN areas as identified based on their conservation value and alignment with the Wildlife Division's priority corridors (MNRT 2022). These corridors (Figure 7) represent key areas for enhancing ecological connectivity, biodiversity conservation, and ES provision. Baseline LULC data for OPAN areas were obtained from the 2022 ESRI LULC dataset. Within corridors (MNRT 2022), degraded areas, including barren land, farmland, and settlements, were identified as potential targets for restoration. Two restoration scenarios were then developed: upper‐bound restoration (100% restoration) and partial restoration (50% restoration), transforming degraded land back to natural habitats. Specifically, in savanna rangeland and flooded savanna ecosystems, degraded areas were restored to grassland LULC type, while in montane forest, coastal forest, miombo woodland and mangroves were restored to forest/woodland LULC type.

The spatial analysis was conducted in QGIS by intersecting degraded LULC with OPAN areas overlapping 53 wildlife corridors to quantify the potential restoration extent. These spatial extents were used to calculate the monetary values of selected ESs under each scenario, following the procedure explained in section 2.3 above. In the upper‐bound scenario, 100% restoration was assumed by 2030; this represents an ecological idealisation, as recovery trajectories are rarely linear and may take decades depending on site conditions, land‐use pressures, and stakeholder engagement. The partial restoration scenario, by contrast, assumes 50% restoration within the same timeframe. Together, these scenarios are best regarded as indicative pathways, illustrating how restoration could contribute to broader conservation targets, including the Kunming–Montreal Global Biodiversity Framework.

## 3 Results

### 3.1 Status of Different LULC Types Across OPAN Areas

**FIGURE 2:**
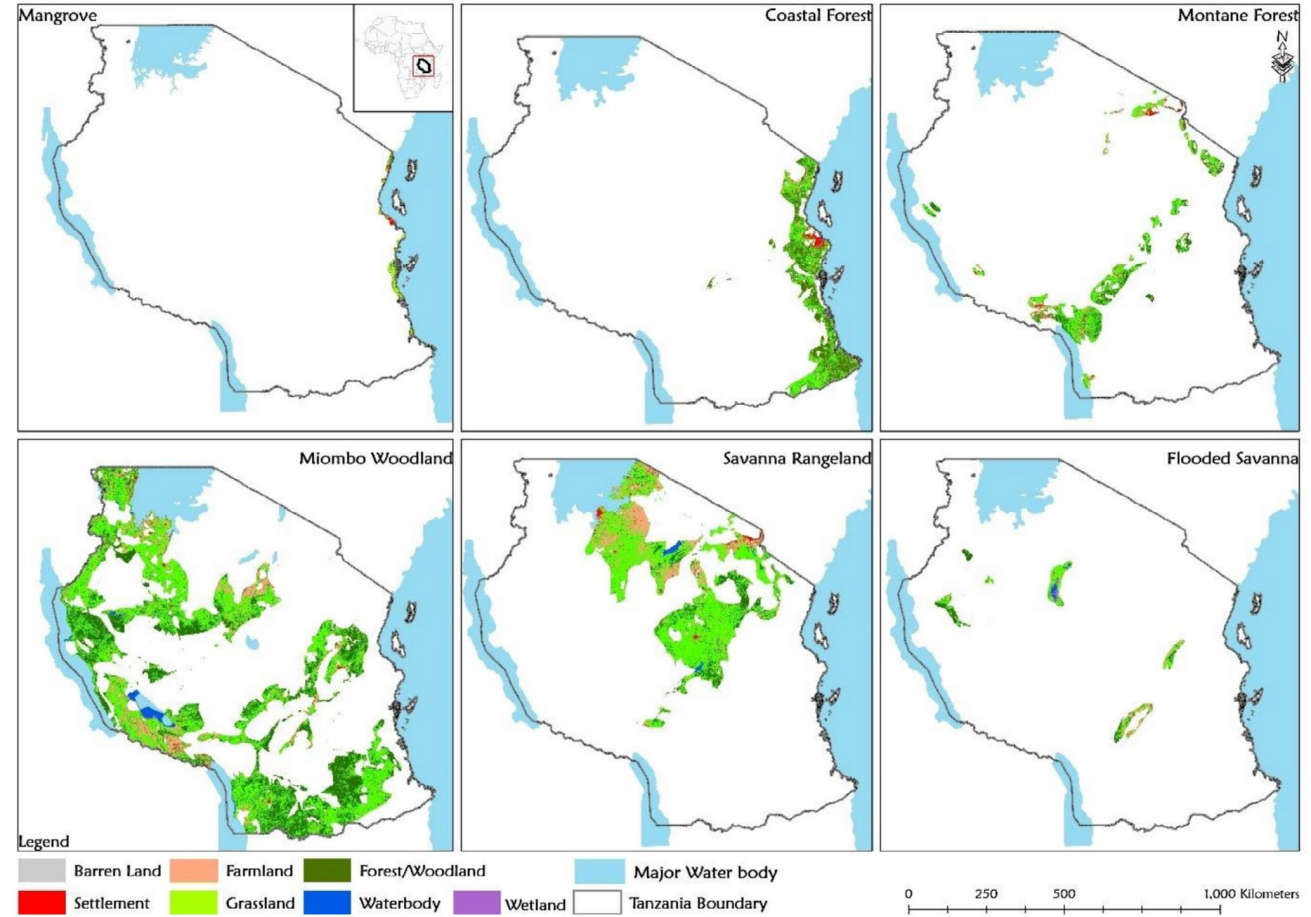
The extent of LULC patch types across OPAN areas in six terrestrial ecosystems in Tanzania.

**FIGURE 3:**
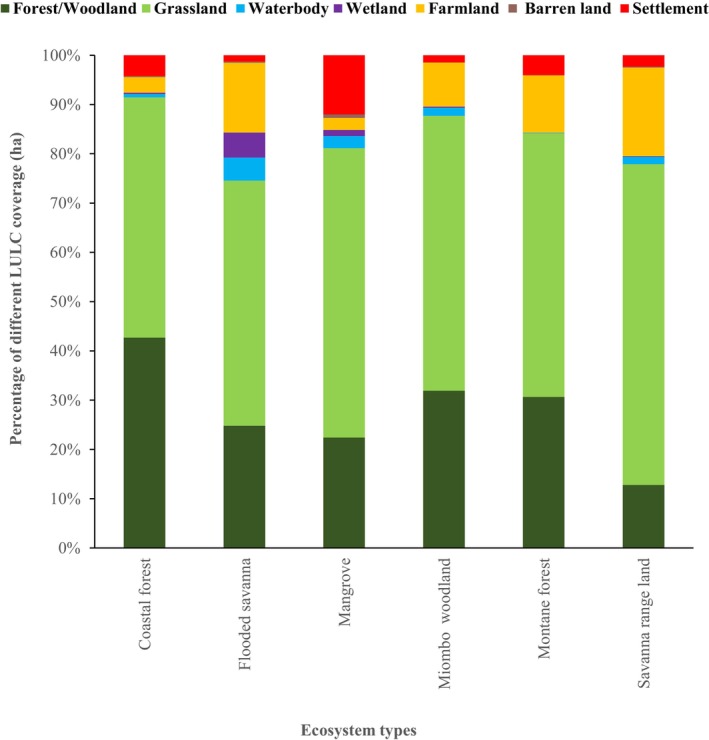
The proportions of the OPAN areas occupied by different LULC types across six terrestrial ecosystems in Tanzania.

Figures 2 and 3 summarise the distribution of LULC types across approximately 55 million ha of OPAN areas within six terrestrial ecosystems. Among ecosystems, miombo woodland had the largest coverage (53%), whereas mangroves accounted for only 1%. Across LULC types (Figure 3), grassland dominated OPAN areas (57%), while barren land covered the smallest proportion (0.1%). At the ecosystem level, grassland notably covered 65% of savanna rangeland and 59% of mangroves, whereas barren land was negligible (< 0.1%) in most ecosystems but slightly higher in mangroves (0.7%).

### 3.2 Monetary Value Estimate of ESs

**FIGURE 4:**
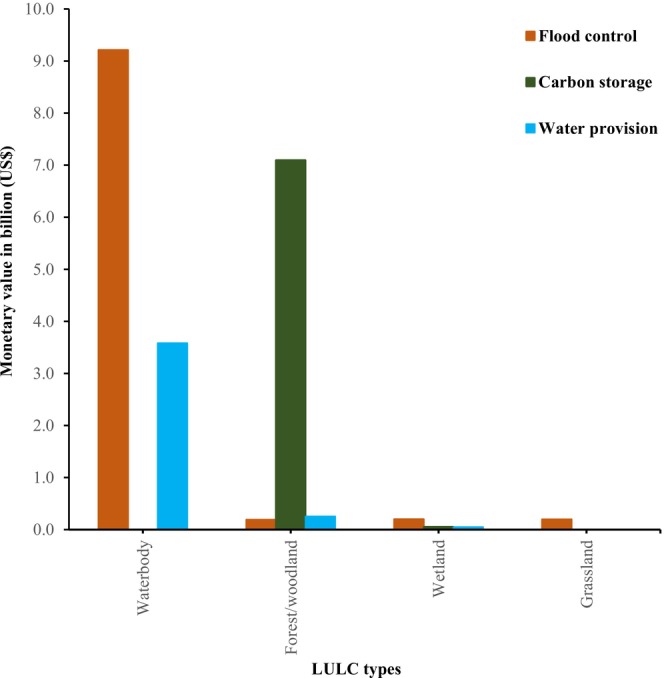
Total annual monetary value estimates of ESs for different LULC types across OPAN areas.

**FIGURE 5:**
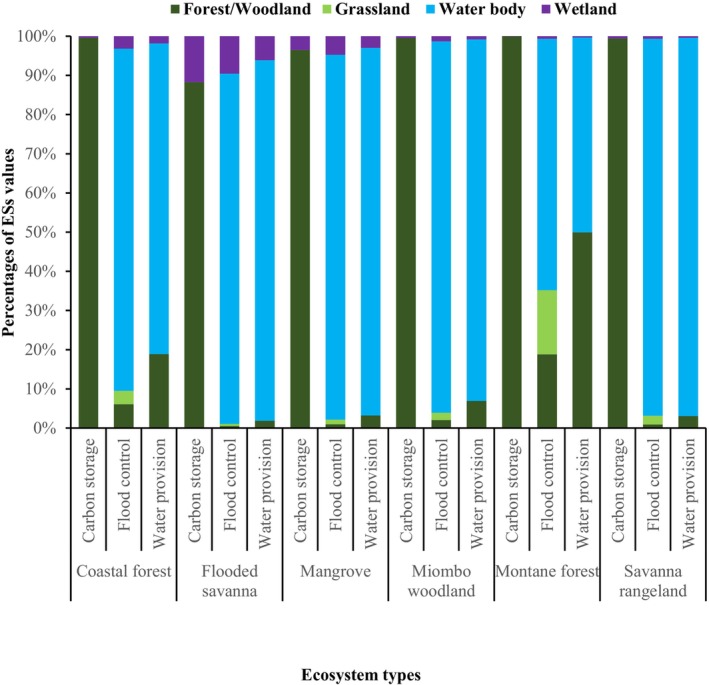
Percentage of annual monetary value estimates of three ESs for four LULC types across OPAN areas for different ecosystems.

**FIGURE 6:**
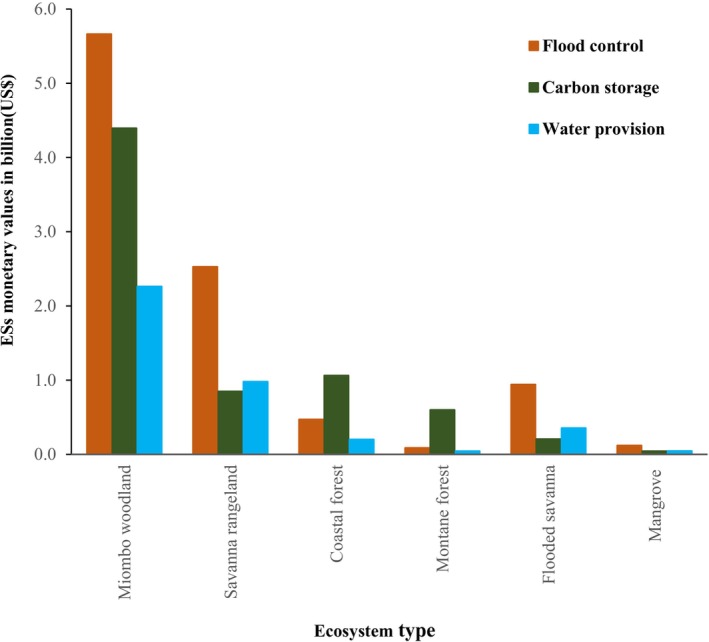
The contribution of each ecosystem to the total annual monetary value of three ecosystem services: flood control, carbon storage, and water provision.

We summarised annual monetary value estimates of three ESs: carbon storage, water provision, and flood control across four LULC types within OPAN areas in six terrestrial ecosystems (Figures 4, 5, 6). The estimated total annual value of the three ESs was US$20.8 billion; 47% from flood control, 34% from carbon storage, and 19% from water provision. The waterbody LULC types yielded the highest estimated ES value, amounting to US$12.8 billion, with contributions of 72% from flood control and 28% from water provision. In contrast, the grassland generated the lowest estimated ES value at US$0.19 billion, with all contributions attributed to flood control (Figure 4). We found that across the OPAN areas, forest/woodland LULC consistently contributed the highest monetary value for carbon storage. Conversely, waterbody coverage was the primary contributor to the monetary value of water provision and flood control ESs (Figure 5). The miombo woodland ecosystem generated the highest monetary value estimate of US$12.4 billion, with flood control contributing 46%. In contrast, the mangrove ecosystem yielded the lowest monetary value, amounting to US$0.2 billion, of which 57% came from flood control (Figure 6).

### 3.3 Monetary Value of ESs in Restored Wildlife Corridors

**FIGURE 7:**
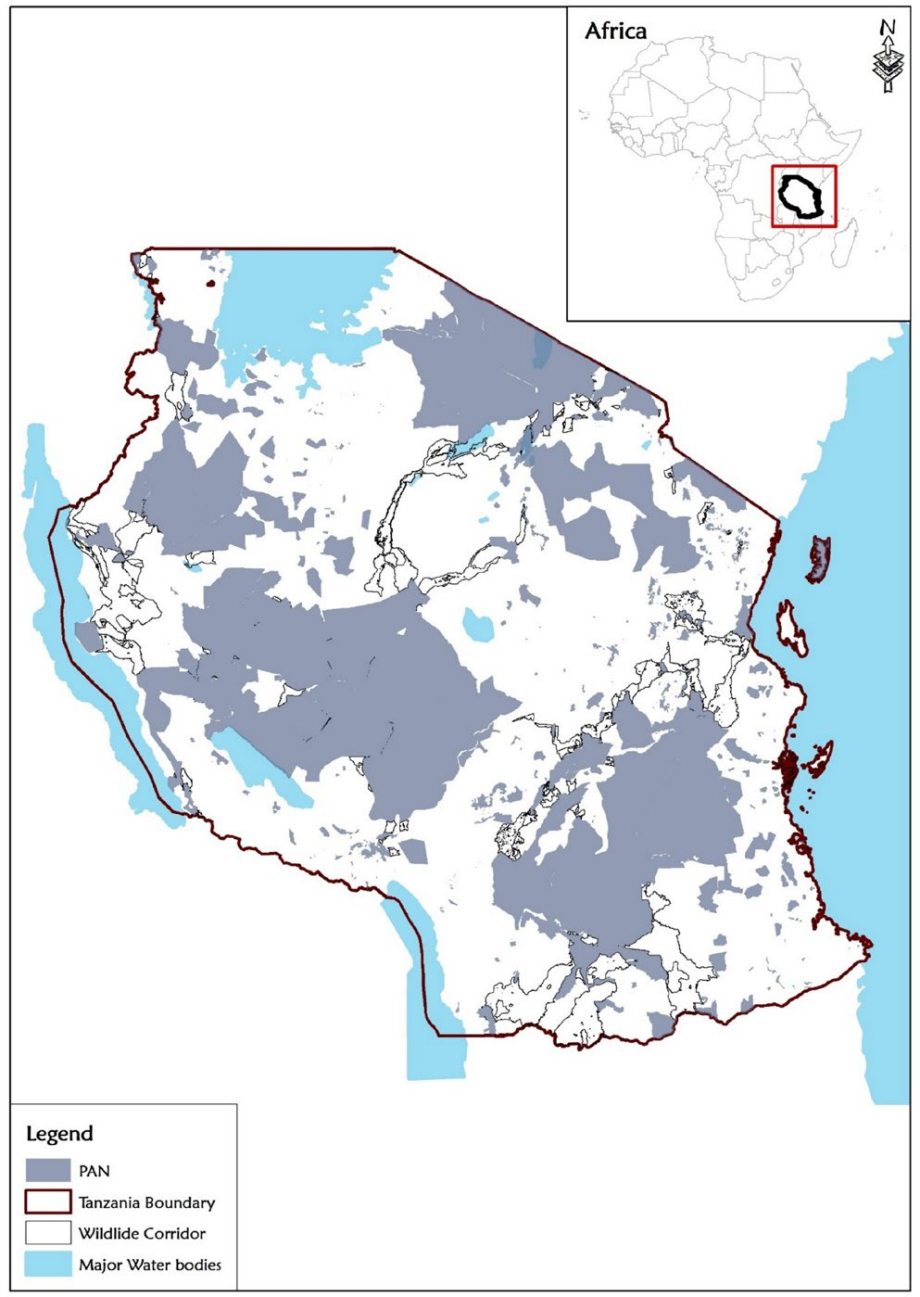
Location of 53 priority wildlife corridors within OPAN areas in Tanzania.

The restoration analysis focused on the 53 prioritised wildlife corridors in Tanzania, covering 22.1% of OPAN areas. Within these corridors, 197,497 ha of degraded land (barren land, farmland, and settlements) were identified as potential areas for restoration to natural habitats (Figure 7). Under 100% restoration, 63.5% of the area was converted to forest/woodland and the remainder to grassland by 2030, generating a total annual monetary value of US$62.8 million in ESs (Table 3). Carbon storage dominated, contributing US$58.7 million (93.4%), entirely from forest/woodland. Water provision accounted for US$2.1 million (3.4%), while flood control contributed US$2.0 million (3.2%), with most benefits (88%) from forest/woodland and the rest from grassland. At 50% restoration (Table 3), both the restored area and associated ES values were reduced by half, yielding a total annual value of US$31.4 million. Carbon storage remained the largest contributor (US$29.3 million), followed by water provision (US$1.1 million) and flood control (US$1.0 million).

**TABLE 3:** Estimated monetary value (US$) of selected ESs across ecosystems under full (100%) and partial (50%).

Ecosystems types	ES type	100% restoration	50% restoration
Savanna rangeland	Carbon storage	—	—
	Water provision	—	—
	Flood control	235,998	117,999
Miombo woodland	Carbon storage	44,966,252	22,483,126
	Water provision	1,613,138	806,569
	Flood control	1,209,856	604,928
Montane forest	Carbon storage	11,540,344	5,770,172
	Water provision	414,002	207,001
	Flood control	310,502	155,251
Coastal forest	Carbon storage	2,069,930	1,034,965
	Water provision	74,258	37,129
	Flood control	55,692	27,846
Flooded savanna	Carbon storage	—	—
	Water provision	—	—
	Flood control	2946	1473
Mangroves	Carbon storage	109,513	54,756
	Water provision	3929	1965
	Flood control	218,736	109,368

## 4 Discussion

Our findings show that OPAN areas have the potential to contribute to ES provisioning, thereby reinforcing their relevance within an EBC framework that extends beyond formal PAs. Detailed spatial analysis of LULC types coupled with monetary assessment of selected ESs reveals that a notable portion of terrestrial ecosystems in Tanzania, despite lacking formal protection, may contribute to ESs provisioning such as carbon storage, water provision and flood control, among others. While these values are indicative, they demonstrate the possible contributions of OPANs in complementing biodiversity conservation and ES provisioning traditionally associated with formal PAs. These services are essential for ecosystem integrity and may also create opportunities for local stewardship where conservation aligns with local community priorities (Platts et al. 2023).

### 4.1 Spatial Coverage of LULC Types in OPAN Areas

The variation in spatial coverage of different LULC types in OPAN areas across various ecosystems is influenced by both the inherent characteristics of the ecosystems and the degree of degradation they have experienced (Anthony et al. 2024; Makwinja et al. 2021; Muche et al. 2023). Extensive grassland coverage in OPAN areas, including within forest‐dominated ecosystems, underscores the ecological implications of LULC changes. We found that in OPAN areas of forest ecosystems, only a third or less remains as forest patches, with the remainder converted to grassland, farmland or settlements. This spatial insight reveals that changes in LULC, such as the conversion of forest/woodland cover to grasslands, farmlands, or settlements (Bullock et al. 2021; Hamunyela et al. 2020; Mekuria et al. 2021; Ntukey et al. 2022) may reduce the capacity of these ecosystems to provide ESs (Hamilton and Friess 2018; Platts et al. 2023; Richards et al. 2020; Worthington et al. 2020). Nevertheless, the potential for ES provisioning can guide restoration practices and support sustainable OPAN management within national and international policies.

### 4.2 Monetary Values of Different ESs Across LULC Types

Spatial–temporal dynamics in the use and distribution of benefits from ecosystems may influence how communities and societies engage in biodiversity conservation and ecosystem management (Fremier et al. 2013; Morariu et al. 2023; Vierros 2017). Assessing these dynamics is challenging, as it is often difficult to convey the value of ESs (Jaligot et al. 2019; Yuan et al. 2024). Quantifying benefits can help communities to visualise the potential value of conserving specific habitats, and demonstrate the range of benefits achievable from their natural capital (Stegarescu and do Rosario Partidario 2014; Vlieg et al. 2022).

The significant contribution of waterbody coverage (Figure 4) to the total monetary values of water provision and flood control services within OPAN areas suggests its importance in sustaining ESs across the six terrestrial ecosystems studied. Waterbody areas are integral to freshwater ecosystems, providing multiple ESs and functioning as transitional systems (Hettiarachchi et al. 2015) within terrestrial ecosystems. Among these, water provision appears to be a key service (Wang et al. 2024). Inland water ecosystems are globally threatened, having lost over half of their extent (Vári et al. 2022), and are highly susceptible to multiple stressors, particularly chemical and physical pressures (Borgwardt et al. 2019). Flood control reduces the risk of damage to agricultural and settlement areas and is largely influenced by land cover type (Chan et al. 2006). In forest ecosystems, including montane, coastal, and mangrove forests, land cover degradation, such as deforestation (Bullock et al. 2021; Richards et al. 2020; Worthington et al. 2020), may reduce flood control capacity (Brookhuis and Hein 2016). In the savanna rangelands, overgrazing by livestock limits flood control potential (Koka et al. 2024; Liniger and Studer 2019; Shem 2010), while permanent and ungrazed grassland supports this ES (Ford et al. 2012; Jankowska‐Huflejt 2006; Milazzo et al. 2023). Flood risk exacerbated by degraded ecosystems can amplify flood frequency and severity (Bradshaw et al. 2007; Netzer et al. 2019; Peptenatu et al. 2020). These patterns suggest that targeted land and water use policies could help maintain biophysical attributes and optimise ES provision in OPAN areas (Sadokpam et al. 2023). Ecosystem‐level conservation planning in OPANs can support local‐scale assessment, monitoring, and the implementation of context‐based conservation policies (Sadokpam et al. 2023; Vári et al. 2022). Assessing ES monetary values alongside human preferences provides a basis for public awareness and may guide sustainable management of water resources (Vári et al. 2022; Wang et al. 2024).

Our findings indicate that the grassland and wetland LULC types contributed the least to the estimated monetary value attributed to carbon storage ES, while forest/woodland LULC within forest ecosystems (miombo woodland, montane, coastal, and mangrove) accounted for over three‐quarters of this value (Figures 4, 5, 6). The results further indicate that the miombo woodland ecosystem exhibits relatively higher total carbon storage value estimates compared to other ecosystems. Miombo woodland shows relatively higher total carbon storage values, likely due to their large coverage in OPAN areas at (53%, Figure 2 and 32% forest/woodland cover, Figure 3), enhancing carbon sequestration potential. These patterns are consistent with previous studies, linking extensive miombo woodland coverage to greater carbon storage capacity (Mauya et al. 2019; Ribeiro et al. 2013).

Globally, forests sequester billions of tons of CO_2_, providing a natural carbon sink (Favero et al. 2020; Jin et al. 2020; Raihan et al. 2019). Replicating this function through artificial means would be extremely costly (Canadell and Raupach 2008). Mature trees demonstrate resilience to increased CO_2_ levels (Norby et al. 2024), offering potential mitigation benefits in a warming world (Schimel et al. 2015; Walker et al. 2014). Despite this, forest/woodland cover especially in tropical forests, face multiple threats, including land‐use change (Ciais et al. 2011). As deforestation and degradation progress, forest/woodland habitats may lose capacity to provide ESs, such as carbon storage (Ojoatre et al. 2024, 2023). In 2022, 4.1 million hectares of tropical primary forest were lost, equivalent to 11 soccer fields per minute (Weisse et al. 2023), highlighting pressures on biodiversity and ESs provisioning. These observations support the use of targeted conservation and restoration strategies in OPAN areas within an EBC framework.

### 4.3 Future LULC Scenarios and Restoration Potential

To ensure sustainable ES provision in OPAN areas, this study highlights the value of conservation and restoration strategies tailored to ecosystem‐specific contexts, with particular emphasis on extending efforts beyond PAN. The future scenario presented in this study (Table 3) highlights the transformative potential of targeted restoration efforts within 53 prioritised wildlife corridors (Table 3) in OPAN areas. This aligns with the Global Biodiversity Conservation Framework target 2, which calls for the effective restoration of at least 30% of degraded terrestrial ecosystems (Global Biodiversity Framework 2022). In our analysis, restoring LULC types within wildlife corridors, currently covering only 1.6% of OPAN areas, was associated with an estimated additional ES value of about US$62.8 million. This suggests that enhancing restoration in OPAN areas may improve ES delivery and increase their economic value, while acknowledging trade‐offs with local livelihoods. To effectively restore forest ecosystems, it is important to prioritise ecosystem‐specific indigenous tree species in grassland patches resulting from forest degradation, as these species are adapted to local conditions and ecological interactions (Bond et al. 2019). This approach can maintain biodiversity, expand the total area of natural habitats (Norton et al. 2018) and support ESs such as carbon storage and water provision (Elliott et al. 2023; Song et al. 2023). In savanna rangeland and flooded savanna ecosystems, maintaining native grassland species and avoiding their misinterpretation as degraded land is essential to prevent inappropriate restoration interventions (Bond et al. 2019; Vetter 2020). Regulating grazing intensity and limiting the conversion to farmlands or settlements (Otieno and Kinyamario 2018; Zerga 2015) would further support these ecosystems. Barren lands in savanna ecosystems also present opportunities for restoration by promoting the regrowth of native grassland species. Restoring natural grass cover with indigenous herbaceous plants can enhance biodiversity conservation and improve ESs, such as soil carbon storage, water provision, and flood control (Vetter 2020). Wetlands and waterbodies, which have undergone some of the most severe degradation (Schmitz 2012; Vári et al. 2022), should be prioritised in restoration planning. Natural regeneration through removal of anthropogenic pressures and allowing ecosystems to recover offers one pathway (Schmitz 2012). While this process is often slow and depends on community willingness for support, it may yield long‐term ecological benefits. Restoration efforts whether active or natural, could enhance ES provision across ecosystems particularly in carbon storage, water regulation and flood control. At the same time, restoration must account for human land‐use needs. In this study, the proposed restoration was to degraded areas within priority wildlife corridors, where ecological connectivity and species movement are under pressure. Even so, restoring farmland or settlement land to native vegetation could potentially affect local livelihoods and resource access. These trade‐offs underscore the importance of integrating ecological goals with inclusive land‐use planning and participatory decision‐making consistent with an EBC framework.

### 4.4 Ecosystem‐Based Conservation Framework for OPAN Areas Management

Overall, our findings suggest that OPAN areas can contribute to ES provision outside formal PAs, complementing conservation within PAN. Spatial analyses, monetary valuations, and restoration scenarios collectively indicate that these unprotected areas have the potential to sustain carbon storage, water provision, and flood control, particularly where forest/woodland cover and waterbodies remain intact. Although OPANs are not formally protected, their management can benefit from the EBC framework, which integrates ES provisioning, biodiversity conservation, and restoration priorities at the ecosystem level. By applying an EBC framework, high‐value areas for specific ES and targeted conservation or restoration strategies can be implemented. This approach highlights synergies between services provided in PAN and OPAN areas, supporting broader landscape‐level conservation outcomes. For instance, Buschke and Capitani (2023) reported higher ES benefits in some OPAN compared to PAN areas, suggesting the complementary role of these areas. Policies should therefore explicitly integrate OPANs within the EBC framework, aligning with Tanzania's National Biodiversity Strategy and Action Plan, the National Land Use Policy, and other relevant national strategies, as well as global initiatives for the integrated management of shared landscapes (Toomey 2020). Assigning monetary values to ESs can help create incentives, encourage community engagement in conservation and restoration efforts and raise awareness of these areas (Benra et al. 2022; Thapa et al. 2022; Wang et al. 2024). Framing OPAN management within the EBC framework provides a practical pathway to link local ES provisioning, including gains from ecological restoration, with national and global biodiversity goals, including the Kunming‐Montreal Global Biodiversity Framework (Global Biodiversity Framework 2022). By integrating spatial, ecological, and socio‐economic evidence, this synthesis highlights how OPAN areas, despite being unprotected, can make meaningful contributions to ecosystem resilience and sustainable land management.

## 5 Conclusion

This study highlights the significant role of unprotected areas in ES delivery and underscores the value of incorporating these areas into national and global conservation planning. Using the benefit transfer method, we provided indicative estimates of ES values across different LULC types in OPAN areas, which can inform restoration and land‐use planning. The restoration scenarios demonstrate how targeted interventions in prioritised wildlife corridors could enhance ES provision and contribute to global conservation targets. For example, restoring 1.6% degraded land within Tanzania's wildlife corridors was estimated to generate US$62.8 million in ES value, largely from carbon storage. These results suggest that OPAN areas can play a meaningful role in advancing the Kunming–Montreal Global Biodiversity Framework's target for ecosystem restoration. Notably, this study offers an adaptable and practical conservation approach for integrating OPAN management into the EBC framework. By linking ecological integrity with human well‐being, this approach supports the adoption of land‐use practices that balance restoration with local development needs and long‐term sustainability.

## Author Contributions

**Florence Godfrey Tarimo:** conceptualization (equal), data curation (lead), formal analysis (lead), funding acquisition (lead), investigation (lead), methodology (equal), project administration (lead), resources (lead), validation (lead), visualization (equal), writing – original draft (lead), writing – review and editing (equal). **Francis Moyo:** methodology (supporting), supervision (supporting), visualization (supporting), writing – review and editing (equal). **Claire Kelly:** methodology (supporting), supervision (supporting), visualization (supporting), writing – review and editing (equal). **Linus Kasian Munishi:** conceptualization (equal), data curation (supporting), formal analysis (supporting), methodology (equal), project administration (supporting), resources (supporting), visualization (equal), investigation (supporting), supervision (lead), validation (supporting), writing – original draft (supporting), writing – review and editing (equal).

## Conflicts of Interest

The authors declare no conflicts of interest.

## Supporting information


**Table S1:** Description of different land use land cover types in the study area.

**Table S2:** Ecosystem service types across different ecosystems.

## Data Availability

All data supporting the findings of this study are included in the main manuscript and Supporting Information submitted with this article.
