# Supplementary material for: Conservation Planning for Promoting Ecosystem Service Provisioning Outside Protected Area Networks
Source: Ecol Evol. 2025 Nov 29;15(12):e72576. doi: 10.1002/ece3.72576 (PMC12663744; doi:10.1002/ece3.72576)
Supplement: Supplementary file 1 — Table S1: Description of different land use land cover types in the study area. [file ECE3-15-e72576-s001.pdf]

Table S1. Description of different land use land cover types in the study area

LULC type	*Description*
Barren Land	Land areas of exposed soil surfaces as influenced by human impacts and/or natural causes
Settlement	Land areas which are characterized by a high percentage (30% or more) of constructed materials.
Farmland	Land dominated by herbaceous vegetation (75%–100% cover), intensively managed for food production. This includes cultivated fields and agroforestry systems with crops as the primary component, excluding pastures and natural grasslands.
Grassland	Land areas characterised by natural or semi-natural herbaceous vegetation; herbaceous vegetation accounts for 75% to 100% of the cover.
Forest/woodland	Land areas with trees > 6m tall/ areas characterised by tree cover (natural or semi-natural woody vegetation, generally more than 6 meters tall); tree canopy accounts for 25% to 100% of the cover.
Waterbody	Inland areas that are covered with water most of the year, including rivers and lakes
Wetland	Vegetated land with a high-water table and inundated vegetation.

Definitions adapted and modified from (Lobora et al., 2017) and National Land Cover Database (Consortium, 2023; Dewitz, 2021)
