# Supplementary material for: Conservation Planning for Promoting Ecosystem Service Provisioning Outside Protected Area Networks
Source: Ecol Evol. 2025 Nov 29;15(12):e72576. doi: 10.1002/ece3.72576 (PMC12663744; doi:10.1002/ece3.72576)
Supplement: Supplementary file 2 — Table S2: Ecosystem service types across different ecosystems. [file ECE3-15-e72576-s002.pdf]

Table S2. Ecosystem service types across different ecosystems

Ecosystem type	Ecosystem services types
Savanna rangeland	Food, Plant medicine, wood (timber, fuel, and bark), minerals, water, recreation and cultural services, biodiversity maintenance, soil stability, hydrological balance, climate stability (carbon sequestration) (Kaur, 2006); Soil nutrient maintenance, water quality regulation, carbon storage and greenhouse gas mitigation, biodiversity maintenance, Invasion resistance (non-native), agricultural production (forage and livestock) (Guo et al., 2023)
Coastal forest	Carbon sequestration, Climate regulation, erosion control, pharmaceuticals, protection against wind and storms, recreation, tourism, water quality (Ligate et al., 2017); Biomass production, habitat provisioning services, pollination, seed dispersal, resistance to wind storms, fire regulation and mitigation, pest regulation of native and invading insects, carbon sequestration, cultural services, water supply and purification (Brockerhoff et al., 2017); Wood, fisheries, coastal protection, scenic beauty, carbon sequestration and storage, habitat (Arkema et al., 2023); Biodiversity maintenance, nursery ground for fish species (Su et al., 2021)
Mangrove	Nutrient cycling, Nursery and breeding ground, Biomass production, Habitat (Terrestrial and marine fauna), Reducing Eutrophication, Food Products, Fuel Wood, Timber Products, Charcoal Production, Medicines, Fresh Water, Fishing and Aquaculture practices, Water

	Transport, Construction Materials, Climate Regulation and mitigation, Coastal protection, Sequester and store carbon, Flood protection, Storm protection, Wastewater bioremediation, Prevention of saltwater intrusion, Tourism or Eco-Tourism, Nature-based Recreation, Aesthetic value, Cultural Amenities, Education (Bimrah et al., 2022)
Montane forests	Agriculture, nature-based Tourism, Timber, Non-timber products, carbon sequestration, drinking water (Platts et al., 2023); Biomass production, habitat provisioning services, pollination, seed dispersal, resistance to wind storms, fire regulation and mitigation, pest regulation of native and invading insects, Carbon sequestration, Cultural services, Water supply and purification, (Brockerhoff et al., 2017); Climate regulation, flood regulation, game and wild collected food, forage (Schmidt et al., 2019); agricultural, potable water supply, carbon sequestration, recreation and sporting services (Allen et al., 2016).
Miombo woodland	Wild foods, construction materials, fuel, commercial timber harvesting, nutrient cycling, soil erosion regulation, hydrological services, carbon storage and cycling, biodiversity maintenance, cultural services (Ryan et al., 2016)
Flooded savanna	Soil nutrient maintenance, water quality regulation, climate mitigation, biodiversity maintenance, invasion resistance, agricultural production (Guo et al., 2023); Supporting biodiversity, storing carbon, recharging groundwater and removing pollutants, water sources (Mandishona & Knight, 2022)
